# The relationship between migraine and Hashimoto’s thyroiditis: a single center experience

**DOI:** 10.3389/fneur.2024.1370530

**Published:** 2024-02-15

**Authors:** Magdalena Nowaczewska, Marcin Straburzyński, Grzegorz Meder, Marta Waliszewska-Prosół

**Affiliations:** ^1^Athleticomed—Pain and Sport Injury Center with Headache and Migraine Treatment Division, Bydgoszcz, Poland; ^2^Department of Otolaryngology, Head and Neck Surgery, and Laryngological Oncology, Ludwik Rydygier Collegium Medicum in Bydgoszcz, Nicolaus Copernicus University in Toruń, Bydgoszcz, Poland; ^3^Department of Family Medicine and Infectious Diseases, University of Warmia and Mazury, Olsztyn, Poland; ^4^Department of Interventional Radiology, Jan Biziel University Hospital, Bydgoszcz, Poland; ^5^Department of Neurology, Wroclaw Medical University, Wroclaw, Poland

**Keywords:** migraine, headache, outcome, hypothyroiditis, autoimmune thyroiditis, chronification

## Abstract

**Introduction:**

Hashimoto’s thyroiditis (HT) is nowadays the leading cause of hypothyroidism with high and still growing prevalence in general population, but there are lack of data regarding migraine and HT connection.

**Methods:**

The aim of this study was to analyze the prevalence of HT in migraine and to check if the presence of HT influence migraine severity. This retrospective observational cohort study involved consecutive migraine patients consulted at our Headache Center with diagnosis of migraine. Electronic charts of patients were collected, including data on migraine type, presence of cranial autonomic symptoms (CAS), monthly migraine days (MMD), medication overuse headache (MOH), and the presence of comorbidities including HT.

**Results:**

We found 928 eligible migraine patients, 88.7% were women. The mean age was 36.09 years. 592 (63.8%) were diagnosed with episodic migraine (EM), the rest with chronic migraine (CM). MOH was additionally diagnosed in 258 (27.8%) patients. The duration of migraine was 15.99 years. 106 (11.4%) was diagnosed with HT, 148 (15.9%) with hypothyroidisms, while 84 (9.05%) had both diagnosis. Migraine patients with HT were significantly older (*p* < 0.001), were more frequently women (*p* = 0.0017), had longer duration of migraine (*p* < 0.001), had CAS more frequently (<0.001), developed CM (*p* = 0.0169) and depression more frequently (*p* = 0.0047) and had more MMD (*p* = 0.0195) as compared with individuals without HT. According to our multivariate logistic model, the presence CM was positively associated with HT (OR 1.76, *p* = 0.045), MOH and duration of migraine, while negatively associated with aura.

**Conclusion:**

HT is very prevalent in migraine patients. This is the first study considering migraine and HT to be comorbid and suggesting that HT may influence the course of migraine causing its chronification.

## 1 Introduction

Migraine has been linked to several comorbidities, including psychiatric, cardiovascular, hormonal, and pain disorders and thyroid diseases (1, 2). Previous studies found a bidirectional relationship between migraine and hypothyroidism, suggesting that thyroid dysfunction influences migraine and vice versa, and both diseases can be considered to be comorbid (3–5). However, the nature of the relationship remains unclear. The prevalence of hypothyroidism and subclinical hypothyroidism in migraine is higher than that in the general population, as migraine sufferers were found to have a 41% increased risk of developing hypothyroidism (3, 6, 7). Filipchuk et al. found that treated hypothyroidism was significantly more prevalent in chronic migraine compared to episodic migraine patients; thus, it may be associated with migraine chronification (8). Dev et al. demonstrated that treatment of subclinical hypothyroidism effectively reduces migraine headaches (9). Lastly, a genetic study found strong evidence for a genetic correlation between migraine and thyroid dysfunction (hypothyroidism and hyperthyroidism) and identified a shared genetic basis underlying migraine and thyroid traits, meaning that migraine risk is significantly correlated with thyroid disorders, and this relationship is complex and causal (10). On the other hand, the leading cause of hypothyroidism today is Hashimoto’s thyroiditis (HT), an autoimmune thyroid disorder (AITD) characterized by an increased thyroid volume, lymphocyte infiltration of parenchyma, and the presence of specific antibodies. The frequency of HT has considerably increased in recent years, and today, it is one of the most common thyroid diseases (11). The clinical presentation of HT includes three phases, starting with thyrotoxicosis, where stored thyroid hormones are released to blood from destroyed thyroid follicles; next is euthyroidism, where the preserved thyroid tissue compensates for destroyed thyrocytes; finally, there is hypothyroidism, where the production of the thyroid hormone is insufficient. Also, there are studies suggesting that a similar age of onset, remission, sex-specific prevalence, and imbalanced T-cell immune status may place migraine as an autoimmune disease (12, 13). Furthermore, systemic autoimmune diseases, such as rheumatoid arthritis, systemic lupus erythematosus, antiphospholipid syndrome, Sjogren’s syndrome, and psoriasis are more frequent in migraine patients suggesting an association between these pathologies (1, 12). Although hypothyroidism itself is a known cause of headache (classified as headache attributed to a disorder of homeostasis—code 10.4—in the third edition of the International Classification of Headache Disorders 3 (ICHD-3)), one of the migraine comorbidities, as well as a factor linked with migraine chronification, so far, little is known about the relationship between HT and migraine. Although the prevalence of HT in the general population is high and still growing, there is a lack of data regarding migraine and HT connection. Hence, we found only one work that examined the presence of different types of headaches in HT patients and one regarding the prevalence of HT in migraine patients (14, 15). Because of this, and based on our clinical observations, we aimed to retrospectively analyze the prevalence of Hashimoto’s thyroiditis among migraine sufferers visiting our specialized headache clinic considering the clinical characteristics of patients with this disease, including migraine severity.

## 2 Materials and methods

This retrospective observational cohort study involved consecutive migraine patients consulted at our Headache Center in a 3-year period between December 2019 and March 2023. Patients were included in this cohort analysis if they had a diagnosis of migraine with or without aura according to the International Classification of Headache Disorders (ICHD-3) (16). Data from the baseline visits and control visits were extracted from 2019 through November 2021 from the electronic medical database. At the baseline visit, all patients underwent a detailed history-taking and clinical evaluations according to the standard protocol developed in our center. Data on migraine onset age, migraine type, pain location, type of pain, presence of additional migraine symptoms (nausea, vomiting, and photo and phonophobia), monthly migraine days (MMD), monthly headache days (MHD), acute medication days (AMD), the presence of medication overuse headache (MOH), type of acute medication used/overused, headache intensity using a numerical scale (numeric rating scale, NRS), headache burden using the migraine-related disability (Migraine Disability Assessment MIDAS test), number of previous preventive classes failures, responsiveness to triptans, onabotulinumtoxin A, monoclonal antibodies against CGRP (mAbs), and topiramate, family history of migraine, comorbidities, and concomitant medications were collected. Pain was considered unilateral fixed (side-locked) if it occurred on the same side of the head for more than 90% of migraine attacks, unilateral variable if it was unilateral but changed side between attacks or during an attack, or bilateral in all other cases. We considered the patient as having hypothyroidism if a diagnosis had been made by an endocrinologist and the patient was treated with a stable dose of levothyroxine; we excluded patients with a history of hypothyroidism if they did not require L-thyroxine supplementation at the time of examination. We considered a patient as having Hashimoto’s thyroiditis if the diagnosis was made by an endocrinology specialist on the basis of a thyroid ultrasound and a high level of serum thyroid peroxidase antibody, as recommended (11, 17). To confirm a diagnosis, we required a copy of medical history from endocrinology consultation or other medical documentation with a clear HT diagnosis based on ultrasound and antibodies results. The data about other comorbidities like depression, anxiety, hypertension, asthma or autoimmune diseases were taken from the patient history collected over a medical interview. We have some missing data in our sample, regarding minor variables. First is the information about Covid-19 infection, as we started to collect this data since 2021r. Moreover, we have missing data about caffeine intake and menstrual migraine. Also, as not all of our patients undergo neuroimaging, we have missing data regarding brain MRI. Besides, not all of our patients were treated with triptans, mAbs, topiramate or onabotulinumtoxin A, so we have missing data in the treatment area. Our study was approved by the Local Ethics Committee of the Ludwik Rydygier Collegium Medicum in Bydgoszcz. Specific written consent was not required for this retrospective study.

### 2.1 Statistics

For continuous data, descriptive statistics were used to describe the characteristics of the study group: mean, median, standard deviation (SD), first and third quartile values (Q1–Q3) and range (minimum and maximum value). In the case of categorical data, the frequency distribution of individual responses was presented using the counts of each category and their distribution expressed as percentages. These results are shown in the tables. Q-Q plots were used to check if continuous variables follow a normal distribution. Statistical tests used in the study were: U Mann–Whitney test for continuous independent variables and the chi-square test or Fisher’s exact test for independent categorical variables. The U Mann–Whitney test is a non-parametric test used to compare numerical variables between two groups of observations. Statistically significant results, obtained on its basis, indicate the presence of a difference in the distribution of a variable between the groups. When preparing our multivariate logistic model for identifying independent predictors chronic migraine, we considered independent variables, selected from the database. From those factors (independent variables), an optimal set of parameters was selected to build a regression model. The process of selecting the optimal set of prognostic factors was performed using a backward stepwise regression, starting with the model with all potential prognostic factors and eliminating irrelevant variables in subsequent steps minimalizing Akaike Information Criterion (AIC). As a result of the analysis, several parameters were chosen. No adjustment for multiple comparisons were made, as we had only one final model which is not connected to another one and, it was chosen using backwards stepwise regression with AIC, not based on significance level. However, adjustment for multiple comparison was made for exploratory tests using Bejamini-Hochberg method with 15% false discovery rate.

Effect sizes were calculated using V-Cramer’s coefficient for categorical data, and r coefficient for U Mann–Whitney tests for numerical data. In the case of this analysis, the level of statistical significance was set to *p* = 0.05. All calculations were done in *R (version 4.0.2)*.

## 3 Results

We found 928 eligible migraine patients, and 88.7% were women. The mean age was 36.09 ± 10.37 years (range: 18 to 71 years). In total, 592 (63.8%) were diagnosed with episodic migraine (EM), while 336 (33.6%) patients were diagnosed with chronic migraine (CM). Further, 156 (16.8%) patients were diagnosed with migraine with aura. MOH was diagnosed additionally to migraine in 258 (27.8%) patients. The duration of migraine was 15.99 years (range 0.3 to 55 years). In addition, 11.4% (*n* = 106) were diagnosed with HT, 15.9% (*n* = 148) with hypothyroidism, and 9.05% (*n* = 84) had both diagnoses. Only six had hyperthyroidism. Thyroid diseases were the most frequent migraine comorbidity, followed by depression, anxiety and hypertension. As acute treatment, patients were using/overusing mostly triptans, combination codeine medications, and non-steroidal anti-inflammatory drugs (NSAID). Migraine patients with HT were predominantly older (39.56 vs. 35.64 years, *p* < 0.001), had a longer duration of migraine (19.68 vs. 15.52 years, *p* < 0.001), and were more predominantly women (97.2% vs. 87.6%, *p* < 0.0017) as compared with migraine patients without HT. They developed CM and depression more frequently and had more MMD as compared with individuals without HT. CAS were significantly more prevalent in HT group (*p* < 0.001). Individuals with migraine and HT drank more caffeine and had a history of COVID-19 infection more frequently than migraine sufferers without HD. There were no differences in the presence of MOH between both groups. Migraine patients without HT responded significantly more frequently to topiramate, while there were no differences in the response to triptans, mAbs and onabotulinumtoxin A between groups. We also did not find any differences regarding MRI findings between groups. The full characteristics of the patients depending on the presence of HT are presented in Table 1.

**Table 1:** Clinical characteristics of migraine patients depending on the presence of Hashimoto’s thyroiditis (HT).

Variable	Parameter	Migraine with HT (*N* = 106)	Migraine without HT (*N* = 822)	*p*-value	Effect size
Sex	Woman	97.2% (*N* = 103)	87.6% (*N* = 720)	0.0017^a^	0.096
	Man	2.8% (*N* = 3)	12.4% (*N* = 102)		
Age (years)	Mean (SD)	39.56 (9.64)	35.64 (10.38)	<0.001^a^	0.124
Duration of migraine (years)	Mean (SD)	19.68 (11.35)	15.52 (10.34)	<0.001^a^	0.118
Type of migraine	Episodic	52.8% (*N* = 56)	65.2% (*N* = 536)	0.0169^a^	0.082
	Chronic	47.2% (*N* = 50)	34.8% (*N* = 286)		
Migraine with aura	Visual	12.3% (*N* = 13)	12.5% (*N* = 103)	0.8385	
	Complex	2.8% (*N* = 3)	4.5% (*N* = 37)		
	No	84.9% (*N* = 90)	83% (*N* = 682)		
Menstrual migraine/ menstrually related migraine	Yes	25% (*N* = 17)	18% (*N* = 86)	0.2238	
	No	75% (*N* = 51)	82% (*N* = 392)		
Additional migraine symptoms	One	16% (*N* = 17)	14.1% (*N* = 116)	0.3562	
	Two	36.8% (*N* = 39)	33.2% (*N* = 273)		
	Three	37.7% (*N* = 40)	36.7% (*N* = 302)		
	Four	9.4% (*N* = 10)	15.9% (*N* = 131)		
CAS	Yes	24.5% (*N* = 26)	8% (*N* = 66)	<0.001^a^	0.176
	No	75.5% (*N* = 80)	92% (*N* = 756)		
Pulsating type of pain	Yes	63.2% (*N* = 67)	65.3% (*N* = 536)	0.7534	
	No	36.8% (*N* = 39)	34.7% (*N* = 285)		
Localization of pain	Bilateral	37.7% (*N* = 40)	32.7% (*N* = 268)	0.4533	
	Unilateral (variable side)	21.7% (*N* = 23)	26.6% (*N* = 218)		
	Unilateral (fixed side)	40.6% (*N* = 43)	40.7% (*N* = 334)		
Additional types of pain	Yes	6.6% (*N* = 7)	10.9% (*N* = 90)	0.2272	
	No	93.4% (*N* = 99)	89.1% (*N* = 732)		
MOH	Yes	32.1% (*N* = 34)	27.3% (*N* = 224)	0.3572	
	No	67.9% (*N* = 72)	72.7% (*N* = 597)		
Triptan responders	Yes	73.9% (*N* = 34)	72.5% (*N* = 237)	0.9777	
	No	26.1% (*N* = 12)	27.5% (*N* = 90)		
mAbs responders	Yes	75% (*N* = 12)	71.8% (*N* = 74)	1	
	No	25% (*N* = 4)	28.2% (*N* = 29)		
Botulinum toxine BoNT-A responders	Effective	75% (*N* = 3)	57.6% (*N* = 19)	0.6328	
	Ineffective	25% (*N* = 1)	42.4% (*N* = 14)		
Topiramate responders	Yes	63.6% (*N* = 7)	32.1% (*N* = 25)	0.0512	
	No	36.4% (*N* = 4)	67.9% (*N* = 53)		
Prior preventive classes failures	0	75.7% (*N* = 56)	72.5% (*N* = 356)	0.3686	
	1	14.9% (*N* = 11)	18.1% (*N* = 89)		
	2	4.1% (*N* = 3)	6.9% (*N* = 34)		
	3	2.7% (*N* = 2)	1% (*N* = 5)		
	> = 4	2.7% (*N* = 2)	1.4% (*N* = 7)		
Acute medication used/overused – Triptan	Yes	29.2% (*N* = 31)	27.4% (*N* = 225)	0.7713	
	No	70.8% (*N* = 75)	72.6% (*N* = 597)		
Acute medication used/overused – Codeine	Yes	20.8% (*N* = 22)	20.6% (*N* = 169)	1	
	No	79.2% (*N* = 84)	79.4% (*N* = 653)		
Acute medication used/overused – NSAID	Yes	32.1% (*N* = 34)	32.6% (*N* = 268)	1	
	No	67.9% (*N* = 72)	67.4% (*N* = 554)		
Acute medication used/overused – Mixed	Yes	1.9% (*N* = 2)	4.4% (*N* = 36)	0.3015	
	No	98.1% (*N* = 104)	95.6% (*N* = 786)		
MMD [days]	N	106	822	0.0195^a^	0.077
	Mean (SD)	9.17 (5.69)	7.94 (5.43)		
	Median (Q1–Q3)	8 (5–15)	7 (4–10)		
	Range	0.5–30	0.1–30		
MHD [days]	N	106	822	0.1572	
	Mean (SD)	13.49 (9.35)	12.22 (9.22)		
	Median (Q1–Q3)	12 (5.25–20)	10 (5–20)		
	Range	0–30	0–30		
AMD [days]	N	106	822	0.082	
	Mean (SD)	11.23 (8.79)	9.73 (8.14)		
	Median (Q1–Q3)	8 (5–16)	7 (4–15)		
	Range	0–30	0–30		
NRS	N	106	822	0.5488	
	Mean (SD)	8.63 (1.3)	8.56 (1.27)		
	Median (Q1–Q3)	9 (8–10)	8 (8–10)		
	Range	5–10	4–10		
MIDAS	N	106	808	0.5248	
	Mean (SD)	57.73 (51.42)	52.72 (47.1)		
	Median (Q1–Q3)	36.5 (23–78.25)	38 (19–74)		
	Range	2–215	1–254		
MIDAS – severity	little or no disability (0–5)	4.7% (*N* = 5)	10% (*N* = 81)	0.2749	
	mild disability (6–10)	7.5% (*N* = 8)	5% (*N* = 40)		
	moderate disability (11–20)	9.4% (*N* = 10)	11.2% (*N* = 90)		
	severe disability (21–40)	32.1% (*N* = 34)	27.6% (*N* = 223)		
	very severe disability (41–270)	46.2% (*N* = 49)	46.2% (*N* = 373)		
Depression	Yes	23.6% (*N* = 25)	12.9% (*N* = 106)	0.0047^a^	0.098
	No	76.4% (*N* = 81)	87.1% (*N* = 716)		
Anxiety	Yes	8.5% (*N* = 9)	6% (*N* = 49)	0.4241	
	No	91.5% (*N* = 97)	94% (*N* = 773)		
Oral contraceptives	Yes	14.2% (*N* = 15)	16.2% (*N* = 133)	0.692	
	No	85.8% (*N* = 91)	83.8% (*N* = 689)		
MRI pathology	Hyperintensive signals	23.2% (*N* = 13)	16.4% (*N* = 51)	0.3714	
	Cysts	8.9% (*N* = 5)	6.1% (*N* = 19)		
	Other	8.9% (*N* = 5)	8% (*N* = 25)		
	No	58.9% (*N* = 33)	69.5% (*N* = 216)		
Family history of migraine	Yes	58.5% (*N* = 62)	55.2% (*N* = 454)	0.5949	
	No	41.5% (*N* = 44)	44.8% (*N* = 368)		
Autoimmune diseases	Yes	4.7% (*N* = 5)	2.6% (*N* = 21)	0.2068	
	No	95.3% (*N* = 101)	97.4% (*N* = 801)		
Asthma/ allergy	Yes	1.9% (*N* = 2)	2.1% (*N* = 17)	1	
	No	98.1% (*N* = 104)	97.9% (*N* = 805)		
Chronic Vertigo	Yes	2.8% (*N* = 3)	1.2% (*N* = 10)	0.1778	
	No	97.2% (*N* = 103)	98.8% (*N* = 812)		
Hypertension	Yes	8.5% (*N* = 9)	4.9% (*N* = 40)	0.1804	
	No	91.5% (*N* = 97)	95.1% (*N* = 782)		
Caffeine intake [cups]	N	70	488	0.0435^a^	0.082
	Mean (SD)	1.75 (1.38)	1.44 (1.36)		
	Median (Q1–Q3)	1.5 (1–2)	1 (0–2)		
	Range	0–6	0–10		
	No	64.7% (*N* = 22)	73.4% (*N* = 138)		
History of Covid-19 infection	Yes	63.4% (*N* = 26)	46.6% (*N* = 110)	0.0491^a^	0.079
	No	36.6% (*N* = 15)	53.4% (*N* = 126)		
History of multiple Covid-19 infection	Yes	23.1% (*N* = 6)	9.1% (*N* = 10)	0.0985	
	No	76.9% (*N* = 20)	90.9% (*N* = 100)		

a Statistically significant after Benjamini-Hochberg adjustment.

HT, Hashimoto’s thyroiditis; CAS, cranial autonomic symptoms; MOH, medication overuse headache; mAbs, monoclonal antibodies; NSAID, non steroidal anti-inflammatory drugs; MMD, monthly migraine days; MHD, monthly headache days; AMD, acute medication days; NRS-numeric rating scale; MIDAS, Migraine Disability Assessment Test; MRI, magnetic resonance imaging.

All the CM patients with HT were women, while the prevalence of males in the CM without HT group was 10.8% (*p* < 0.0075). Patients with CM and HT developed bilateral localization of pain mire frequently than individuals with CM without HT (Table 2). The MMD in the migraine with hypothyroidism group were not statistically different from those in the migraine without hyperthyroidism group (8.48 vs. 8; *p* < 0.2965), but they used triptans and had history of multiple Covid-19 infections more frequently (Table 3).

**Table 2:** Clinical characteristics of chronic migraine (CM) patients depending on the presence of Hashimoto’s thyroiditis (HT).

Variable	Parameter	Chronic migraine with HT (*N* = 50)	Chronic migraine without HT (*N* = 286)	*p*-value	Effect size
Sex	Woman	100% (*N* = 50)	89.2% (*N* = 255)	0.0075^a^	0.080
	Man	0% (*N* = 0)	10.8% (*N* = 31)		
Age [years]	N	50	286	0.0454	
	Mean (SD)	41.4 (10.04)	37.87 (10.3)		
	Median (Q1–Q3)	41.5 (35–47)	39 (30–45)		
	Range	25–71	17–68		
Duration of migraine [years]	N	50	286	0.04	
	Mean (SD)	22.56 (12.13)	18.7 (10.61)		
	Median (Q1–Q3)	20.5 (15–30)	18 (10–27)		
	Range	2–50	1–55		
Type of migraine	Episodic	0% (*N* = 0)	0% (*N* = 0)	1	
	Chronic	100% (*N* = 50)	100% (*N* = 286)		
Migraine with aura	Visual	10% (*N* = 5)	10.1% (*N* = 29)	1	
	Complex	0% (*N* = 0)	1% (*N* = 3)		
	No	90% (*N* = 45)	88.8% (*N* = 254)		
Menstrual migraine	Yes	14.7% (*N* = 5)	13.1% (*N* = 22)	0.7847	
	No	85.3% (*N* = 29)	86.9% (*N* = 146)		
Additional migraine symptoms	One	22% (*N* = 11)	14.3% (*N* = 41)	0.1255	
	Two	36% (*N* = 18)	31.5% (*N* = 90)		
	Three	36% (*N* = 18)	37.1% (*N* = 106)		
	Four	6% (*N* = 3)	17.1% (*N* = 49)		
CAS	Yes	20% (*N* = 10)	10.5% (*N* = 30)	0.0931	
	No	80% (*N* = 40)	89.5% (*N* = 256)		
Pulsating type of pain	Yes	56% (*N* = 28)	66% (*N* = 188)	0.231	
	No	44% (*N* = 22)	34% (*N* = 97)		
Localization of pain	Bilateral	50% (*N* = 25)	32.4% (*N* = 92)	0.0306	
	Unilateral (variable side)	14% (*N* = 7)	27.5% (*N* = 78)		
	Unilateral (fixed side)	36% (*N* = 18)	40.1% (*N* = 114)		
Additional types of pain	Yes	0% (*N* = 0)	0.3% (*N* = 1)	1	
	No	100% (*N* = 50)	99.7% (*N* = 285)		
MOH	Yes	66% (*N* = 33)	71.3% (*N* = 204)	0.5522	
	No	34% (*N* = 17)	28.7% (*N* = 82)		
Triptan responders	Yes	59.1% (*N* = 13)	73.9% (*N* = 116)	0.2322	
	No	40.9% (*N* = 9)	26.1% (*N* = 41)		
mAbs responders	Yes	60% (*N* = 6)	72% (*N* = 54)	0.4708	
	No	40% (*N* = 4)	28% (*N* = 21)		
Botulinum toxine BoNT-A responders	Effective	66.7% (*N* = 2)	65.4% (*N* = 17)	1	
	Ineffective	33.3% (*N* = 1)	34.6% (*N* = 9)		
Topiramate responders	Yes	60% (*N* = 6)	31.2% (*N* = 15)	0.1456	
	No	40% (*N* = 4)	68.8% (*N* = 33)		
Prior preventive classes failures	0	73% (*N* = 27)	56.5% (*N* = 100)	0.1816	
	1	13.5% (*N* = 5)	26.6% (*N* = 47)		
	2	5.4% (*N* = 2)	11.9% (*N* = 21)		
	3	5.4% (*N* = 2)	2.8% (*N* = 5)		
	> = 4	2.7% (*N* = 1)	2.3% (*N* = 4)		
Acute medication used/overused – Triptan	Yes	26% (*N* = 13)	42% (*N* = 120)	0.0486	
	No	74% (*N* = 37)	58% (*N* = 166)		
Acute medication used/overused – Codeine	Yes	42% (*N* = 21)	34.6% (*N* = 99)	0.3978	
	No	58% (*N* = 29)	65.4% (*N* = 187)		
Acute medication used/overused – NSAID	Yes	30% (*N* = 15)	29.7% (*N* = 85)	1	
	No	70% (*N* = 35)	70.3% (*N* = 201)		
Acute medication used/overused – Mixed	Yes	4% (*N* = 2)	12.6% (*N* = 36)	0.0908	
	No	96% (*N* = 48)	87.4% (*N* = 250)		
MMD [days]	N	50	286	0.8246	
	Mean (SD)	13.42 (5.03)	13.33 (4.9)		
	Median (Q1–Q3)	15 (9.25–16)	13.5 (9–16)		
	Range	8–30	6–30		
MHD [days]	N	50	286	0.5952	
	Mean (SD)	21.64 (5.83)	22.07 (6.14)		
	Median (Q1–Q3)	20 (16–30)	20 (16–30)		
	Range	16–30	0–30		
AMD [days]	N	50	286	0.7837	
	Mean (SD)	17.98 (8.19)	17.8 (7.82)		
	Median (Q1–Q3)	16 (10.5–23)	16 (12–25)		
	Range	4–30	0–30		
Other headache [days]	N	50	286	0.3995	
	Mean (SD)	7.92 (6.92)	8.84 (7.14)		
	Median (Q1–Q3)	7.5 (0–14.75)	8 (2–15)		
	Range	0–22	0–25		
NRS	N	50	286	0.2791	
	Mean (SD)	8.82 (1.19)	8.6 (1.3)		
	Median (Q1–Q3)	9 (8–10)	9 (8–10)		
	Range	6–10	5–10		
MIDAS	N	50	284	0.5745	
	Mean (SD)	87.76 (58.97)	92.36 (54.31)		
	Median (Q1–Q3)	77.5 (32.5–142.25)	87 (45–136.25)		
	Range	2–215	2–254		
MIDAS – severity	little or no disability (0–5)	2% (*N* = 1)	2.1% (*N* = 6)	0.1925	
	mild disability (6–10)	8% (*N* = 4)	3.2% (*N* = 9)		
	moderate disability (11–20)	4% (*N* = 2)	3.2% (*N* = 9)		
	severe disability (21–40)	20% (*N* = 10)	13.1% (*N* = 37)		
	very severe disability (41–270)	66% (*N* = 33)	78.4% (*N* = 222)		
Depression	Yes	28% (*N* = 14)	18.5% (*N* = 53)	0.1757	
	No	72% (*N* = 36)	81.5% (*N* = 233)		
Anxiety	Yes	8% (*N* = 4)	8.7% (*N* = 25)	1	
	No	92% (*N* = 46)	91.3% (*N* = 261)		
Thyroid disease	Hypothyroidism	74% (*N* = 37)	7% (*N* = 20)	<0.001^a^	0.274
	Hyperthyroidism	2% (*N* = 1)	0.3% (*N* = 1)		
Oral contraceptives	Yes	14% (*N* = 7)	16.4% (*N* = 47)	0.8231	
	No	86% (*N* = 43)	83.6% (*N* = 239)		
MRI pathology	Hyperintensive signals	25% (*N* = 7)	19.1% (*N* = 25)	0.2548	
	Cysts	14.3% (*N* = 4)	6.1% (*N* = 8)		
	Other	10.7% (*N* = 3)	8.4% (*N* = 11)		
	No	50% (*N* = 14)	66.4% (*N* = 87)		
Family history of migraine	Yes	56% (*N* = 28)	58.7% (*N* = 168)	0.8358	
	No	44% (*N* = 22)	41.3% (*N* = 118)		
Autoimmunological diseases	Yes	4% (*N* = 2)	3.5% (*N* = 10)	0.6953	
	No	96% (*N* = 48)	96.5% (*N* = 276)		
Asthma/ allergy	Yes	2% (*N* = 1)	1.4% (*N* = 4)	0.5555	
	No	98% (*N* = 49)	98.6% (*N* = 282)		
Vertigo	Yes	6% (*N* = 3)	1% (*N* = 3)	0.0448	
	No	94% (*N* = 47)	99% (*N* = 283)		
Hypertension	Yes	10% (*N* = 5)	7.3% (*N* = 21)	0.5639	
	No	90% (*N* = 45)	92.7% (*N* = 265)		
Caffeine intake [cups]	N	36	173	0.0967	
	Mean (SD)	1.89 (1.51)	1.51 (1.55)		
	Median (Q1–Q3)	2 (1–3)	1 (0–2)		
	Range	0–6	0–10		
	No	64.7% (*N* = 11)	71% (*N* = 44)		
History of Covid-19 infection	Yes	57.9% (*N* = 11)	50.6% (*N* = 39)	0.7567	
	No	42.1% (*N* = 8)	49.4% (*N* = 38)		
History of multiple Covid-19 infection	Yes	36.4% (*N* = 4)	5.1% (*N* = 2)	0.0166	
	No	63.6% (*N* = 7)	94.9% (*N* = 37)		

a Statistically significant after Benjamini-Hochberg adjustment.

HT, Hashimoto’s thyroiditis; CAS, cranial autonomic symptoms; MOH, medication overuse headache; mAbs, monoclonal antibodies; NSAID, non steroidal anti-inflammatory drugs; MMD, monthly migraine days; MHD, monthly headache days; AMD, acute medication days; NRS-numeric rating scale; MIDAS, Migraine Disability Assessment Test; MRI, magnetic resonance imaging.

**Table 3:** Clinical characteristics of migraine patients depending on the presence of hypothyroidism.

Variable	Parameter	Migraine with hypothyroidism (*N* = 148)	Migraine without hypothyroidism (*N* = 780)	*p*-value	Effect size
Sex	Woman	98% (*N* = 145)	86.9% (*N* = 678)	<0.001^a^	0.128
	Man	2% (*N* = 3)	13.1% (*N* = 102)		
Age [years]	N	148	780	0.0064^a^	0.090
	Mean (SD)	38.43 (10.8)	35.64 (10.23)		
	Median (Q1-Q3)	38 (30–45.25)	35 (28–43)		
	Range	17–71	14–68		
Duration of migraine [years]	N	148	780	0.0146^a^	0.080
	Mean (SD)	18.04 (11.03)	15.6 (10.4)		
	Median (Q1-Q3)	18 (10–25.25)	14 (7–22)		
	Range	1–50	0.3–55		
Type of migraine	Episodic	61.5% (*N* = 91)	64.2% (*N* = 501)	0.5867	
	Chronic	38.5% (*N* = 57)	35.8% (*N* = 279)		
Migraine with aura	Visual	12.2% (*N* = 18)	12.6% (*N* = 98)	0.9751	
	Complex	4.1% (*N* = 6)	4.4% (*N* = 34)		
	No	83.8% (*N* = 124)	83.1% (*N* = 648)		
Menstrual migraine	Yes	19.8% (*N* = 20)	18.7% (*N* = 83)	0.8998	
	No	80.2% (*N* = 81)	81.3% (*N* = 362)		
Additional migraine symptoms	One	10.8% (*N* = 16)	15% (*N* = 117)	0.3307	
	Two	33.1% (*N* = 49)	33.7% (*N* = 263)		
	Three	42.6% (*N* = 63)	35.8% (*N* = 279)		
	Four	13.5% (*N* = 20)	15.5% (*N* = 121)		
CAS	Yes	25% (*N* = 37)	7.1% (*N* = 55)	<0.001^a^	0.220
	No	75% (*N* = 111)	92.9% (*N* = 725)		
Pulsating type of pain	Yes	64.9% (*N* = 96)	65.1% (*N* = 507)	1	
	No	35.1% (*N* = 52)	34.9% (*N* = 272)		
Localization of pain	Bilateral	40.5% (*N* = 60)	31.9% (*N* = 248)	0.0597	
	Unilateral (variable side)	19.6% (*N* = 29)	27.2% (*N* = 212)		
	Unilateral (fixed side)	39.9% (*N* = 59)	40.9% (*N* = 318)		
Additional types of pain	Yes	11.5% (*N* = 17)	10.3% (*N* = 80)	0.7627	
	No	88.5% (*N* = 131)	89.7% (*N* = 700)		
MOH	Yes	25.7% (*N* = 38)	28.2% (*N* = 220)	0.5903	
	No	74.3% (*N* = 110)	71.8% (*N* = 559)		
Triptan responders	Yes	79.7% (*N* = 63)	70.7% (*N* = 208)	0.1468	
	No	20.3% (*N* = 16)	29.3% (*N* = 86)		
mAbs responders	Yes	80% (*N* = 16)	70.7% (*N* = 70)	0.5847	
	No	20% (*N* = 4)	29.3% (*N* = 29)		
Botulinum toxine responders	Effective	71.4% (*N* = 5)	56.7% (*N* = 17)	0.6767	
	Ineffective	28.6% (*N* = 2)	43.3% (*N* = 13)		
Topiramate responders	Yes	41.2% (*N* = 7)	34.7% (*N* = 25)	0.8276	
	No	58.8% (*N* = 10)	65.3% (*N* = 47)		
Prior preventive classes failures	0	72.9% (*N* = 78)	72.9% (*N* = 334)	0.9064	
	1	17.8% (*N* = 19)	17.7% (*N* = 81)		
	2	5.6% (*N* = 6)	6.8% (*N* = 31)		
	3	1.9% (*N* = 2)	1.1% (*N* = 5)		
	> = 4	1.9% (*N* = 2)	1.5% (*N* = 7)		
Acute medication used/overused – Triptan	Yes	35.8% (*N* = 53)	26% (*N* = 203)	0.0192^a^	0.080
	No	64.2% (*N* = 95)	74% (*N* = 577)		
Acute medication used/overused – Codeine	Yes	16.9% (*N* = 25)	21.3% (*N* = 166)	0.2712	
	No	83.1% (*N* = 123)	78.7% (*N* = 614)		
Acute medication used/overused – NSAID	Yes	33.8% (*N* = 50)	32.3% (*N* = 252)	0.7982	
	No	66.2% (*N* = 98)	67.7% (*N* = 528)		
Acute medication used/overused – Mixed	Yes	1.4% (*N* = 2)	4.6% (*N* = 36)	0.0708	
	No	98.6% (*N* = 146)	95.4% (*N* = 744)		
MMD [days]	N	148	780	0.2965	
	Mean (SD)	8.48 (5.51)	8 (5.47)		
	Median (Q1-Q3)	8 (4–10.5)	7 (4–10)		
	Range	0.5–30	0.1–30		
MHD [days]	N	148	780	0.3792	
	Mean (SD)	12.74 (9.08)	12.29 (9.27)		
	Median (Q1–Q3)	10 (6–20)	10 (5–20)		
	Range	0–30	0–30		
AMD [days]	N	148	780	0.4106	
	Mean (SD)	10.16 (8.1)	9.85 (8.25)		
	Median (Q1–Q3)	8 (5–15)	7 (4–15)		
	Range	0–30	0–30		
NRS	N	148	780	0.1534	
	Mean (SD)	8.7 (1.31)	8.55 (1.27)		
	Median (Q1–Q3)	9 (8–10)	8 (8–10)		
	Range	5–10	4–10		
MIDAS	N	146	768	0.6113	
	Mean (SD)	51.99 (47.97)	53.55 (47.58)		
	Median (Q1–Q3)	36 (22.25–59)	38 (19–75)		
	Range	2–254	1–243		
MIDAS – severity	little or no disability (0–5)	6.8% (*N* = 10)	9.9% (*N* = 76)	0.0962	
	mild disability (6–10)	6.2% (*N* = 9)	5.1% (*N* = 39)		
	moderate disability (11–20)	9.6% (*N* = 14)	11.2% (*N* = 86)		
	severe disability (21–40)	37% (*N* = 54)	26.5% (*N* = 203)		
	very severe disability (41–270)	40.4% (*N* = 59)	47.3% (*N* = 363)		
Depression	Yes	18.9% (*N* = 28)	13.2% (*N* = 103)	0.0888	
	No	81.1% (*N* = 120)	86.8% (*N* = 677)		
Anxiety	Yes	9.5% (*N* = 14)	5.6% (*N* = 44)	0.1154	
	No	90.5% (*N* = 134)	94.4% (*N* = 736)		
Hashimoto	Yes	56.8% (*N* = 84)	2.8% (*N* = 22)	<0.001^a^	0.621
	No	43.2% (*N* = 64)	97.2% (*N* = 758)		
Oral contraceptives	Yes	16.9% (*N* = 25)	15.8% (*N* = 123)	0.8262	
	No	83.1% (*N* = 123)	84.2% (*N* = 657)		
MRI pathology	Hyperintensive signals	16.9% (*N* = 13)	17.6% (*N* = 51)	0.9656	
	Cysts	7.8% (*N* = 6)	6.2% (*N* = 18)		
	Other	7.8% (*N* = 6)	8.3% (*N* = 24)		
	No	67.5% (*N* = 52)	67.9% (*N* = 197)		
Family history of migraine	Yes	59.5% (*N* = 88)	54.9% (*N* = 428)	0.3474	
	No	40.5% (*N* = 60)	45.1% (*N* = 352)		
Autoimmunological diseases	Yes	3.4% (*N* = 5)	2.7% (*N* = 21)	0.5904	
	No	96.6% (*N* = 143)	97.3% (*N* = 759)		
Asthma/ allergy	Yes	2% (*N* = 3)	2.1% (*N* = 16)	1	
	No	98% (*N* = 145)	97.9% (*N* = 764)		
Vertigo	Yes	1.4% (*N* = 2)	1.4% (*N* = 11)	1	
	No	98.6% (*N* = 146)	98.6% (*N* = 769)		
Hypertension	Yes	6.8% (*N* = 10)	5% (*N* = 39)	0.4992	
	No	93.2% (*N* = 138)	95% (*N* = 741)		
Caffeine intake [cups]	N	103	455	0.9908	
	Mean (SD)	1.43 (1.15)	1.49 (1.41)		
	Median (Q1–Q3)	1 (1–2)	1 (0–2)		
	Range	0–5	0–10		
	No	71.4% (*N* = 35)	72.3% (*N* = 125)		
History of Covid-19 infection	Yes	58.2% (*N* = 32)	46.8% (*N* = 104)	0.1755	
	No	41.8% (*N* = 23)	53.2% (*N* = 118)		
History of multiple Covid-19 infection	Yes	28.1% (*N* = 9)	6.7% (*N* = 7)	0.003^a^	0.108
	No	71.9% (*N* = 23)	93.3% (*N* = 97)		

a Statistically significant after Benjamini-Hochberg adjustment.

HT, Hashimoto’s thyroiditis; CAS, cranial autonomic symptoms; MOH, medication overuse headache; mAbs, monoclonal antibodies; NSAID, non steroidal anti-inflammatory drugs; MMD, monthly migraine days; MHD, monthly headache days; AMD, acute medication days; NRS-numeric rating scale; MIDAS, Migraine Disability Assessment Test; MRI, magnetic resonance imaging.

Given the results presented in Table 1, we decided to prepare a multivariate logistic model to evaluate the factors linked with the presence of chronic migraine. According to that model, MOH (OR: 97.6, *p* < 0,001), the duration of migraine (OR: 1.031, *p* = 0,003), and HT (OR 1.76, *p* = 0.045), were positively associated with migraine chronification, whereas it was negatively associated with the presence of visual and complex aura (Table 4).

**Table 4:** Multivariate logistic model evaluating independent variables associated with the presence of chronic migraine.

Variable	Estimate	OR	LCI	UCI	*p*-value
Intercept	−2.114	0.121	0.078	0.182	<0.001
MOH	4.581	97.569	51.202	208.174	<0.001
Duration of migraine [years]	0.031	1.031	1.010	1.053	0.003
Migraine with aura: Visual	−0.489	0.613	0.299	1.177	0.160
Migraine with aura: Complex	−2.122	0.120	0.017	0.575	0.020
Hashimoto’s thyroiditis	0.568	1.764	0.949	3.186	0.045
Acute medication used/overused – Codeine	0.510	1.665	0.925	2.925	0.082

MOH, medication overuse headache.

## 4 Discussion

To the best of our knowledge, this is the first study to investigate the presence of HT and describe the clinical characteristics of patients with HT in a large, almost one thousand migraine patient cohort. The main finding of our study is a high prevalence of HT among migraine patients. According to our data thyroid diseases are the most frequent migraine comorbidities, as 11.4% of our patients were diagnosed with HT, 15.9% with hypothyroidism, and 9.05% had both diagnoses. Our results prove that migraine patients with HT differ from non HT group as they are significantly older, are more frequently women, had a longer duration of migraine, had CAS and depression more frequently, developed CM more frequently, drank more caffeine and had a history of COVID-19 infection more frequently.

Spanou et al. found that the prevalence of any type of thyroid disorder in the primary headache group was 20.8% (89/427 patients), with 6.3% reporting hypothyroidism and only 2.8% reporting HT (15). Yin et al. discovered that thyroid diseases were more prevalent in the migraine group than in the control group (7.2% vs. 2.8%) (18). Another study evaluated the incidence of primary headache in the HT group and found that 61.3% of cases were diagnosed with headache (21.1% migraine, 17.9% tension-type headaches, and 21.1% new daily persistent headaches) (14). In our group, 11.4% of migraine sufferers were diagnosed with HT, which is higher than the estimated prevalence of HT in the general population (reported as 7.5%) (19). We found very high prevalence of the female sex among HT and migraine patients, as 97.2% were woman, so it was higher than expected. Surprisingly, all patients with CM and HT were woman. This findings might be only partially explained by the fact that migraine is more prevalent in woman. In fact, gender-related differences are more significant in HT patients. According to the latest data the overall prevalence of HT in adults is 7.5%, with a prevalence of 17.5% in women and 6.0% in men, so the risk of developing HT in adult women is approximately 4 times than that of adult men (19). Another older study reported that the ratio of female HT patients to male HT patients was even higher: 8–9:1 (20). Thus, both diseases are more prevalent in females and possible explanations could be found in the role of female sex hormones (11). In addition, migraine and systemic autoimmune diseases are two-to threefold more common in women (12). The fact that HT is an autoimmunological disease may explain the HT and migraine connection. Previous studies show that headache and migraine are more prevalent in systemic autoimmune diseases, and endothelial dysfunction is the alteration that is common among all these disorders (12). HT was also found to be very prevalent in fibromyalgia patients, and similar studies revealed a bidirectional link between fibromyalgia and migraine (21, 22).

The prevalence of CAS in both the HT and hypothyroidism groups was significantly higher than in groups without thyroid diseases in our study. This is the first observation, as we failed to find any existing data linking CAS with thyroid diseases. CAS result from intense peripheral trigeminal activation but also may be triggered by a central sensitization (23). Waliszewska-Prosół et al. aimed to evaluate the parameters of visual and brainstem auditory evoked potentials (VEP, BAEP) in euthyreotic HT patients without central nervous system involvement. They found a significantly higher P100 VEP amplitude in HT in the HT group as compared to the control group, indicating increased bioelectrical activity of the cerebral cortex in those patients (24). Interestingly, a similar phenomenon of excessive bioelectrical cortex activity has been described in migraine patients (25). This activation may lead to cranial pathways sensitization and further migraine chronification. One may not exclude that HT by activating cerebral cortex may influence the increased CAS presence. Both groups of patients, migraine and HT, also showed similar abnormalities in metabolic composition as assessed by MRI spectroscopy of the brain. Decreased levels of N-acetyl-aspartate (NAA) and increased lactate (Lac) were shown in both diseases, which may indicate decreased neuronal activity within the normal appearing brain in patients with HT and migraine (26–28).

Interestingly, we found that CM was significantly more prevalent in the migraine with HT group, as almost half of individuals had this type of disease as compared with the non-HT group. Also, there were significant differences in the MMD between groups (9.17 vs. 7.94, *p* < 0.019). Contrary to other authors, we did not find a difference between the migraine groups with and without hypothyroidism. Our multivariate logistic model found HT, not hypothyroidism, to be an variable associated with the presence of CM. Filipchuk et al. noticed that treated hypothyroidism was significantly more prevalent in CM patients (29.55%) compared to EM patients, thus pointing to hypothyroidism as a risk factor for migraine chronification (8). Starikova et al. found an association of a more severe clinical course of migraine with lower thyroid-stimulating hormone levels (5). On the other hand, some authors demonstrated that migraine may be a result of high thyroid-stimulating hormone (TSH) levels, which can lead to pituitary growth and the compression of intracellular structures (29). Another study found a strong correlation between hypothyroidism, CM, and new daily persistent headache (30). It is worth noting that the presence of HT in the hypothyroidism group as well as in the whole group was not accessed or mentioned in any of these studies. Nevertheless, it is highly possible there were individuals with HT present inside those groups. Whether HT is a consequence or a cause of CM is a matter to be studied and discussed in the future.

In our multivariate logistic model not only HT but also MOH and duration of migraine were positively associated with the presence of CM. The latter two are already know factors causing migraine chronification (31, 32). The presence of aura was negatively associated with CM, which was already noticed by other authors (33).

Caffeine intake was significantly higher in the patients with HT. This was a rather unexpected finding as HT patients on levothyroxine supplementation therapy should avoid concomitant coffee consumption due to the possible impact on drug absorption (34). One study found an inverse association between vitamin D levels and coffee consumption in HT patients (35).

COVID-19 infection was significantly more prevalent in the HT and migraine patients, and current data suggest that COVID-19 may cause autoimmune thyroid disease or exacerbate the underlying thyroid disease in remission (36). The autoimmune process plays a major role in HT etiopathogenesis, although the exact mechanism is not fully understood (37). This immune reaction is mostly limited to the thyroid gland with extrathyroidal manifestations secondary to hypothyroidism or, in rare cases, thyrotoxicosis. The disfunction of regulatory T cells (Treg) is secondary to genetic and environmental factors. In particular, Tregs forming the CD4^+^CD25^+^Foxp3^+^, CD4^+^CD69^+^Foxp^− 2^, and CD4^+^CD49^+^LAG-3^+^IL-10^+^ classes seem to be of importance (38, 39). This regulatory T-cells dysfunction allows thyroid cells injury via cytotoxic and humoral mechanisms (37). However, Treg dysfunction may not be limited to this organ, as patients with HT are at the higher risk of developing other autoimmune disorders (40). Interestingly, the Treg defect may also be associated with migraine, as patients with migraine have lower serum levels of these cells (41). The Tregs activity was also responsible for alleviating trigeminal sensitization in a chronic migraine animal model (42). In that study, low-dose IL-2, a Tregs activator, was used to alleviate trigeminal activation. Apart from these findings, it should be noted that serum levels of IL-17 are elevated in HT (43). Interestingly Il-17 may cross the blood–brain barrier and activate trigeminal nucleus caudalis neurons adding to possible immune mechanisms leading to a positive correlation between migraine and HT (44). However, in our study, we did not find any difference regarding the coexistence of autoimmunological diseases between the migraine and the HT groups.

Although a previous study found significant associations between the presence of white matter hyperintensities and thyroid gland dysfunction, we failed to find any differences in the MRI findings between the migraine with HT and the migraine without HT group (45).

Our study has several limitations, most of them linked to the retrospective design of our study. One is the lack of information regarding the serum thyroid hormone level and the serum thyroid peroxidase antibody level of our patients. Another may be a lack of thyroid ultrasound results, especially in the non-HT group. Also, the data regarding migraine comorbidities including depression, anxiety, HT, asthma or autoimmune disorders was taken from the patient’s history taken by interview. Besides, we did not use the standardized migraine diaries to assess MMD. One of a biggest limitation of our study it its single-center design. Although our sample is large and achieves statistical power, the patients come from the same area, thus study population is more homogeneous than might be expected of a population made up of patients from different clinics. This may produce higher risk of bias linked with small exposure variability, decreased generalizability of the results and small geographical variation of the estimated effects. This may particularly influence the data regarding the prevalence of HT in our cohort, as in our region the serum thyroid antibodies are quite routine and popular tests and HT awareness in our population is very high. Nevertheless, our results build up a rationale to conduct a big prospective multicenter study or genetic study to access the nature of the migraine and HT relationship.

## 5 Conclusion

Hashimoto’s thyroiditis is very prevalent in migraine patients, particularly among woman, and is one of the most frequent migraine comorbidity. This is the first study describing the clinical characteristics of migraine patients with HT. According to our study migraine patients with HT differ from individuals without HT, as they are significantly older, they are more frequently women, had a longer duration of migraine, had CAS and depression more frequently, and developed CM more frequently. Our results allow migraine and HT to be considered comorbid and suggest that HT may influence the course of migraine, causing its chronification.

## Data availability statement

The original contributions presented in the study are included in the article/supplementary material, further inquiries can be directed to the corresponding author.

## Ethics statement

The studies involving humans were approved by Local Ethics Committee of the Ludwik Rydygier Collegium Medicum in Bydgoszcz. The studies were conducted in accordance with the local legislation and institutional requirements. Written informed consent for participation was not required from the participants or the participants’ legal guardians/next of kin because of the type of the study – it was a retrospective study.

## Author contributions

MN: Conceptualization, Data curation, Investigation, Methodology, Project administration, Resources, Writing – original draft, Writing – review & editing. MS: Data curation, Writing – review & editing. GM: Data curation, Writing – review & editing. MW-P: Data curation, Writing – review & editing.
